# Calsequestrins New Calcium Store Markers of Adult Zebrafish Cerebellum and Optic Tectum

**DOI:** 10.3389/fnana.2020.00015

**Published:** 2020-04-21

**Authors:** Sandra Furlan, Marina Campione, Marta Murgia, Simone Mosole, Francesco Argenton, Pompeo Volpe, Alessandra Nori

**Affiliations:** ^1^Consiglio Nazionale delle Ricerche, Institute of Neuroscience, Padova, Italy; ^2^Department of Biomedical Sciences, University of Padova, Istituto Interuniversitario di Miologia, Padova, Italy; ^3^Department of Proteomics and Signal Transduction, Max-Planck-Institute of Biochemistry, Martinsried, Germany; ^4^Institute of Oncology Research (IOR), Bellinzona, Switzerland; ^5^Department of Biology, University of Padova, Padova, Italy

**Keywords:** granule cell, Purkinje cell, calcium stores, Zebrafish, Ca-binding protein

## Abstract

Calcium stores in neurons are heterogeneous in compartmentalization and molecular composition. *Danio rerio* (zebrafish) is an animal model with a simply folded cerebellum similar in cellular organization to that of mammals. The aim of the study was to identify new endoplasmic reticulum (ER) calcium store markers in zebrafish adult brain with emphasis on cerebellum and optic tectum. By quantitative polymerase chain reaction, we found three RNA transcripts coding for the intra-ER calcium binding protein calsequestrin: *casq1a*, *casq1b*, and *casq2*. In brain homogenates, two isoforms were detected by mass spectrometry and western blotting. Fractionation experiments of whole brain revealed that Casq1a and Casq2 were enriched in a heavy fraction containing ER microsomes and synaptic membranes. By *in situ* hybridization, we found the heterogeneous expression of *casq1a* and *casq2* mRNA to be compatible with the cellular localization of calsequestrins investigated by immunofluorescence. Casq1 was expressed in neurogenic differentiation 1 expressing the granule cells of the cerebellum and the periventricular zone of the optic tectum. Casq2 was concentrated in parvalbumin expressing Purkinje cells. At a subcellular level, Casq1 was restricted to granular cell bodies, and Casq2 was localized in cell bodies, dendrites, and axons. Data are discussed in relation to the differential cellular and subcellular distribution of other cerebellum calcium store markers and are evaluated with respect to the putative relevance of calsequestrins in the neuron-specific functional activity.

## Introduction

Zebrafish (*Danio rerio*) is an emerging animal model whose developed brain, cells, and neuronal circuits are similar to those of mammals and thus is suitable to study human brain pathology (Saleem and Kannan, 2018). Calcium (Ca^2+^) storage in neurons is crucial for cell activity and death (Zündorf and Reiser, 2011). Three major compartments are involved in intracellular Ca^2+^ homeostasis: endoplasmic reticulum (ER), mitochondria, and cytosol. The ER Ca^2+^ store is not homogeneous: families of channels, pumps, and storage proteins are key players in the regulation of Ca^2+^ concentration and cycling in specific compartments to ensure integration and/or strictly localized responses to multiple stimuli. The ER Ca^2+^ stores are involved in neuronal physiology by multiple mechanisms. The IP3-sensitive Ca^2+^ stores are involved in neural plasticity and memory formation in mammals (Inoue et al., 1998; Rose and Konnerth, 2001) and are suggested to elevate the resting Ca^2+^ concentration of the pre-synaptic compartment upon Ca^2+^ depletion of the synaptic space. Moreover, the release of Ca^2+^ from IP3-sensitive Ca^2+^ stores has been involved in long-term depression (LTD) mechanisms. Calcium-induced calcium release from ER Ca^2+^ stores has been proposed as the mechanism for the transmission of a Ca^2+^ signal from the periphery to the cell body in order to regulate gene transcription in long-term potentiation [extensively reviewed by Verkhratsky (2005) and Brini et al. (2014)]. The capacity of Ca^2+^ stores depends both on the intra-ER Ca^2+^ binding proteins that provide releasable Ca^2+^ in specific cell regions and the prolonged stimulations. The identification and the localization of Ca^2+^ handling proteins expressed selectively and/or in combination within a specific neuronal class are essential to identify different cells and their Ca^2+^ handling processes during development and in adult tissues. Cytoplasmic neuronal Ca^2+^ binding proteins have been identified in *D. rerio*: for example, parvalbumin 7 (Pvalb7) is selectively expressed in Purkinje cells of the cerebellum and cerebellum-like structures during development and in adult individuals (Bae et al., 2009; Takeuchi et al., 2015), calretinin (29-kDa calbindin or calbindin 2) is expressed in the eurydendroid cells of the cerebellum (Castro et al., 2006), calbindin 2 is a specific marker for granule cells of the LCa area of the cerebellum (Table 1 in Bae et al., 2009), calbindin 1 (D28k) is expressed in ciliate and microvillous cells of the olfactory sensor neurons (Kress et al., 2015), and S100a1 is found in Purkinje cells (Germanà et al., 2008). In addition, different Ca^2+^ binding proteins are expressed in the olfactory bulb and in the spinal cord (Kress et al., 2015; Berg et al., 2018). All these cytoplasmic binding proteins are involved in cytosolic Ca^2+^ buffering during transmission of the action potential.

### Neuronal ER Ca^2+^ Binding Proteins

At rest, the majority of ER Ca^2+^ content is protein bound and rapidly releasable. Intra-ER Ca^2+^ binding proteins are characterized for their low affinity (*K*d ~1 mM) and high capacity for Ca^2+^ (40–80 mol/mol). Calreticulin, expressed in neurons and many other cells, is a multifunctional chaperone involved in protein quality control of secreted proteins. Calsequestrin (Casq) is a key component of sarcoplasmic reticulum Ca^2+^ store in skeletal and in cardiac muscles (Reddish et al., 2017). Casq facilitates Ca^2+^ uptake by sarco/endoplasmic reticulum Ca^2+^-ATPases (SERCA) in the ER by decreasing free intra-ER Ca^2+^concentration. Casq is a peculiar Ca^2+^-binding protein since it has high capacity (from 40 to 80 mol Ca^2+^/mol protein) and low Ca^2+^ affinity (Kd around 1 mM). Binding of Ca^2+^ causes protein oligomerization (Sanchez et al., 2012), allowing strong and dynamic buffering power at low protein concentration. Casq is able to condense at critical sites where ryanodine-sensitive Ca^2+^ release channels are concentrated by specific anchor proteins (Shin et al., 2000). Such a condensation property is missing in other lumenal proteins, widely distributed throughout the ER lumen. Very recently, a role for cardiac Casq in stress response has been proposed (Wang et al., 2019). By and large, these biophysical and physiological properties define and control Ca^2+^ store capacity in the ER of neuronal cells: nonetheless, Casq expression in the brain of fish and of mammals has been poorly investigated. Distinct genes codify different Casq isoforms: in mammals, two genes, *Casq1* and *Casq2* (mouse), encode for two isoforms preferentially expressed in the skeletal muscle and in the heart, respectively. In zebrafish, three genes, *casq1a*, *casq1b*, and *casq2*, are expressed in the skeletal muscle (Furlan et al., 2016) and one (*casq2*) in the cerebellum (Takeuchi et al., 2017). Up to now, Casq2 has been identified as a marker of chicken Purkinje cells only in association with the IP3**-**sensitive Ca^2+^ store and excluded from the ryanodine-sensitive Ca^2+^ store (Volpe et al., 1990, 1991). In zebrafish brain, multiple RNA isoforms of the ryanodine-sensitive Ca^2+^ channel (*Ryr*) have been identified (Darbandi and Franck, 2009; Holland et al., 2017; Tse et al., 2018), but the relative protein products have been partially analyzed (Wu et al., 2011). Zebrafish Purkinje cells express the IP3-sensitive Ca^2+^ channel (ITPR1) in the cell body, the axon, the dendritic shaft, and the spines (Koulen et al., 2000), and adult zebrafish brain proteomic studies have identified Casq2 protein in total homogenates (Nolte et al., 2014; Smidak et al., 2016), but cell-type expression and localization remain to be elucidated. Based on current proteomic and transcriptomic studies, it is evident that multiple isoforms of Ca^2+^ store components are expressed in adult zebrafish brain so that the latter is a suitable model to identify neuronal Ca^2+^ stores in different cells and compartments. A crucial question regards the identification and combination of different molecular markers for a specific Ca^2+^ store and its neuronal-type association. The present article addresses the question pertaining to the expression and the cellular localization of the intra-ER Ca^2+^ binding protein Casq in zebrafish brain. The specific aim of the study is to investigate the differential distribution of Casq isoforms in neurons belonging to the cerebellum and the optic tectum in order to expand knowledge on neuronal Ca^2+^ store markers in zebrafish as a prerequisite to assess their specific functions.

## Materials and Methods

### Ethical Approval

Experiments were carried out on adult (3–6 months) zebrafish under the approval of the University of Padua Ethical Committee on Animal Experimentation and Ministero della Salute (Project Number D2784.N.BGL). The adult fish were maintained and raised in 5-L tanks with freshwater at 28°C, with a 12-h light/12-h dark cycle.

### Quantitative PCR

Total RNA was obtained from pools of adult brains, hearts, and skeletal muscles using TRIzol^®^ extraction method. Reverse transcription, primer sequences, and qPCR are described in Furlan et al. (2016). Normalization was performed by ΔCT method using B2M and EF1alpha as reference genes. Values are expressed as mean (*n* = 3) ± SEM.

### Protein Methods (Preparation of Tissue Extracts, Subcellular Fractionation, Western Blotting, and *in vitro* Deglycosylation)

Whole homogenates were prepared as previously described (Salvatori et al., 1997). Briefly, the tissues were homogenized with a Teflon pestle-equipped Potter-Elvehjem tissue grinder in the presence of a medium containing 3% (wt/vol) SDS, 0.1 mM EGTA, pH 7.0, and a cocktail of protease inhibitors. The homogenates were then boiled for 5 min and clarified at 15,000 *g* for 10 min. The supernatants were used as whole-protein extracts. Subcellular brain fractionation was carried out essentially as described (Furlan et al., 2016). The brains were homogenized in a homogenization buffer (10 mM Hepes-NaOH, pH 7.4, and 0.32 M sucrose) supplemented with a cocktail of protease inhibitors. The total homogenate was centrifuged for 10 min at 950 *g* and the post-nuclear supernatant (S1) was collected and spun again. S2 was saved and combined with S1, centrifuged at 17,000 *g* for 15 min to yield a pellet corresponding to the mitochondrial fraction (P3) and a supernatant (S3). The S3 containing the remaining organelles from the total homogenate was centrifuged at 30,000 *g* for 1 h to yield a high-speed supernatant (S4) and a pellet (P4) enriched in membranes. Protein concentration was estimated by the method of Lowry using bovine serum albumin as a standard. *In vitro* deglycosylation of glycoproteins was performed on 10 μg of P4 sub-fractions obtained from muscle and brain tissues, using N-glycosydase F deglycosylation kit (Roche) according to the manifacturer’s instructions. Sodium dodecyl sulfate-polyacrylamide gel electrophoresis (SDS-PAGE) and Western blot were carried out as previously described.

PA1-913 (CC) is a polyclonal antibody produced using native canine cardiac Casq as immunogen. In zebrafish, it recognizes mostly Casq2 and, at lower intensity, Casq1a and Casq1b. On the contrary, polyclonal MC reacts mostly with the zebrafish skeletal isoforms; in fact, immunizing peptide for C3868 shows homology with zebrafish Casq1a (67% identity) and Casq1b (44% identity) by BLAST-P analysis, but no homology with zebrafish Casq2 and calreticulin, another Ca^2+^-binding protein enriched in brain that shares some properties (molecular weight and isoelectric point) with Casqs.

**Table 1:** 

Antibodies		
Calsequestrin (CC)	PA1-913	Thermo Fisher Scientific
Calsequestrin (MC)	C3868	Sigma–Aldrich
Calreticulin	PA3-900	Thermo Fisher Scientific
Synaptotagmin1/2	105002	Synaptic System
NDUFS3	3459130	Thermo Fisher Scientific
NeuroD1	ab60704	Abcam
Parvalbumin1	MAB1572	Merck Millipore
Serca2	MA3-910	Thermo Fisher Scientific
ITPR 1	Polyclonal D130	(Volpe et al., 1991)
Stim1	D88E10	Cell signaling
Ryanodine receptor1	MA3-925	Thermo Fisher Scientific

### Mass Spectrometry

Zebrafish brain P4 and S4 protein fractions to be analyzed by MS were resolved by SDS-PAGE and stained with Coomassie Brilliant Blue G-250. Following de-staining, gel slices were washed with 50 mm ammonium bicarbonate and shrunk with ethanol. The reduction/alkylation of proteins was performed with 10 mM dithiothreitol and 55 mM iodoacetamide. After two steps of washing with ammonium bicarbonate/ethanol, the gel was dried with ethanol and incubated with 12.5 ng/μl Lys-C in 50 mM ammonium bicarbonate at 4°C for 15 min. The supernatant was then replaced with fresh 50 mM ammonium bicarbonate, and the reaction allowed to proceed overnight at 37°C. The reaction was stopped with 1% (v/v) trifluoroacetic acid, 0.5% (v/v) acetic acid, and 3% (v/v) acetonitrile, and the supernatant recovered. Additional peptide extraction steps were performed with 30% (v/v) acetonitrile and 100% acetonitrile. The supernatants were concentrated and then diluted with 0.5% (v/v) acetic acid, 30% (v/v) acetonitrile, and 1% (v/v) trifluoroacetic acid. The peptides were desalted and concentrated on reverse-phase C18 StageTips (Rappsilber et al., 2003). Reverse-phase chromatography was performed on a Thermo Easy nLC 1000 system connected to a Q Exactive HF mass spectrometer (Thermo Fisher Scientific) through a nanoelectrospray ion source. The peptides were separated on a 50-cm column with an inner diameter of 75 μm packed in house with 1.9 μm C18 resin (Dr. Maisch GmbH). The peptides were eluted with a linear gradient of acetonitrile 0.1% formic acid at a constant flow rate of 250 nl/min. The column temperature was kept at 50°C by an oven (Sonation GmbH). The eluted peptides from the column were directly electrosprayed into the mass spectrometer.

Mass spectra were acquired in a data-dependent mode to automatically switch between full scan MS and up to 15 data-dependent MS/MS scans. The maximum injection time for full scans was 100 ms, with a target value of 3e6 at a resolution of 120,000 at m/*z* = 200. The target values for MS/MS were set to 1e5, with a maximum injection time of 100 ms at a resolution of 15,000 at m/*z* = 200. To avoid repetitive sequencing, the dynamic exclusion of the sequenced peptides was set to 20 s.

The spectra were analyzed using MaxQuant (version 1.6.6.2). Peak lists were searched against the UNIPROT databases for *D. rerio* (release 2019_08) with common contaminants added. The search included carbamidomethylation of cysteines as fixed modification as well as methionine oxidation and N-terminal acetylation as variable modifications. The maximum allowed mass deviation for MS peaks was set to 6 and 20 ppm for MS/MS peaks. The maximum missed cleavages were two. The false discovery rate was determined by searching a reverse database. The maximum false discovery rates were 0.01 both on the peptide and the protein levels. The minimum required peptide length was six residues. Peptide identification was performed with an allowed initial precursor mass deviation of up to 7 ppm and an allowed fragment mass deviation of 20 ppm. Match between runs was used. The mass spectrometry (MS) proteomics data have been deposited to the ProteomeXchange Consortium *via* the PRIDE partner repository with the dataset identifier PXD015577. Bioinformatic analyses were performed with the Perseus software (version 1.5.4.2), part of the MaxQuant environment^1^.

### Immunofluorescence

After sacrifice, the adult zebrafish were quickly peeled to expose the brain in skull, briefly washed in phosphate buffered saline, pH 7.4 (phosphate-buffered saline, PBS), and fixed overnight with 4% paraformaldehyde in PBS. The fixed zebrafish brains were carefully removed from the skull, dehydrated through graded ethanol, and embedded in paraffin as previously described (Moorman et al., 2001). Immunofluorescence analysis was performed on serial 10-μm paraffin wax-embedded sections. After deparaffinization and rehydration, the sections were boiled for 20 min in sodium citrate buffer to induce epitope retrieval (10 mM sodium citrate, 0.05% Tween 20, pH 6.0) and briefly washed in PBS before immunofluorescence assay. The sections were blocked with PBS-Tw-N (PBS, 0.1% Tween20, 5% goat serum) for 30 min to avoid non-specific staining. The sections were then incubated in primary antibodies diluted in PBS-Tw-N for 2–16 h and, after washing, were incubated for 1 h in fluorescently conjugated secondary antibody diluted in PBS-Tw-N. After washing as detailed above, the sections were mounted with ProLong Gold antifade reagent with DAPI (Life Technologies) and cover-slipped. Epi-fluorescence analysis was performed under a Leica DMR microscope using the software Leica Application Suite Advanced Fluorescence 4.0.0.11706 (LASAF). Confocal analysis was performed under Leica SP5 confocal inverted microscope and Zeiss LSM 700 confocal microscope.

### Probe Design

Probes specific for *casq1a* and *casq2* mRNAs were designed by homology search using NCBI-BLAST. Probes were mapped at nucleotides 1,991–2,780 within exon 12 and the 3′ untranslated region for *casq1a* (GenBank accession no. NM_001003620) and at nucleotides 1,038–1,887 within exon 8 and the 3′ untranslated region for *casq2* (GenBank accession no. NM_001002682), respectively. The primers used were *casq1a* (forward primer: 5′-TCCCATTGACCCAATGTTCT-3′, reverse primer: 5′-CCCTTGTGACCAAAGGAAAA-3′, probe size 789 bp) and *casq2* (forward primer: 5′-CGTTTGCTGAAGAGGAGGAC-3′, reverse primer 5′-TGGGTTTTTGCCTTTATTCG-3′, probe size 849 bp). The PCR products were amplified from zebrafish brain cDNA and then cloned into pCR 2.1 and pCR II vectors. Antisense labeled-mRNA was *in vitro* transcribed using digoxygenin-RNA labeling Mix SP6/T7 (Roche Diagnostics GmbH, Mannheim, Germany) following the manufacturer’s instructions.

### *In situ* Hybridization

*In situ* hybridization was performed on serial 10-μm paraffin wax-embedded sections as previously described (Moorman et al., 2001). Briefly, the sections were treated with 20 μg/ml of proteinase K for 15 min at 37°C and postfixed with 4% paraformaldehyde for 20 min. The sections were then washed again before a prehybridization step of 1 h at 70°C with a hybridization solution [50% formamide, 5× SSC, 1% blocking powder (Roche), 0.1% Tween, 0.1% CHAPS, 1 mg/ml yeast tRNA, and 5 mM EDTA]. Next, the fresh solution was added with each of the digoxigenin-labeled RNA probes at the proper dilution. The probes were left to hybridize overnight at 70°C. On the next day, three highly stringent washes were carried out for 30 min each with 50% formamide in SSC. After blocking with B-block for 1 h, the sections were incubated with 1:2,000 sheep anti-digoxigenin alkaline phosphatase-conjugated antibody (Roche) in blocking solution overnight, and the reaction was revealed by BCIP/NBT as substrates.

## Results

### Expression and Immunological Identification of Casqs in Adult Brain

**Figure 1:**
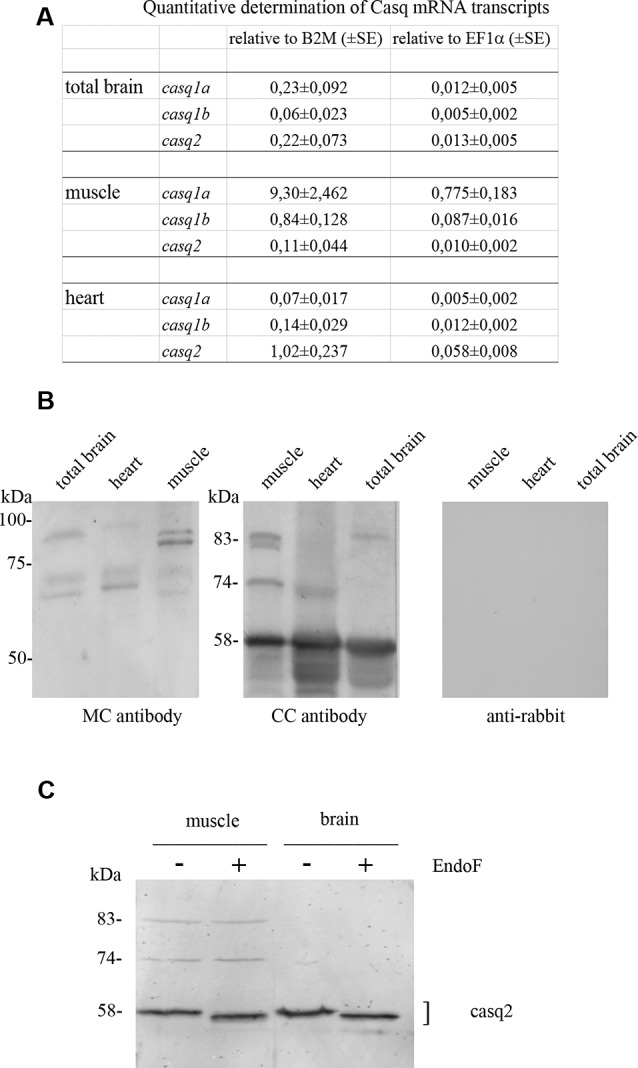
Casq mRNA in selected tissues from adult zebrafish **(A)**. The levels of RNA expression are relative to two different housekeeping genes, beta-2-microglobulin (B2M) and elongation factor1-α (EF1α). Mean values and standard error were determined from triplicate runs of the qPCR assay in three different tissue preparations. **(B)**. Anti-Casq antibodies reactivity on total homogenates (40 μg per lane) obtained from pooled (N = 3) skeletal muscle, heart, and brain of adult zebrafish. In the control lanes, (anti-rabbit) blot incubation was performed in the presence of anti-rabbit conjugate with alkaline phosphatase only. Molecular mass markers (in kDa) are indicated. **(C)** *In vitro* deglycosylation of Casqs. Immunodetection of Casq by CC antibody in membrane-enriched fraction P4 (see “Materials and Methods” section) after endoglycosidase F digestion, with 10 μg loaded per lane.

The relative levels of three transcripts (*casq1a*, *casq1b*, and *casq2*) were measured by qPCR, comparing three tissues (brain, skeletal muscle, and heart). As shown in Figure 1A, the higher expression of *casq1a* was found in the muscle, whereas *casq2* was the most expressed isoform in the heart. In the brain, both *casq1a* and *casq2* were expressed, the lowest expression being observed for *casq1b*.

In order to identify Casqs at the protein level, equal amounts of crude extracts from the skeletal muscle, the heart, and the brain were separated by gel electrophoresis and analyzed by specific antibodies. Two commercially available antibodies, Pa1-913 (CC) and C3868 (MC) raised against mammalian Casqs, recognize zebrafish homologs with different specificity (see Furlan et al., 2016 and below). As shown in Figure 1B, CC antibody mostly reacted with a 58-kDa protein, previously identified as Casq2 in zebrafish skeletal and cardiac muscle. In the brain, a 58-kDa band co-migrating with Casq2 was clearly detected. In the skeletal muscle, the same antibody recognized Casq1 isoforms (doublet at 83 kDa and single band at 74 kDa) in addition to Casq2 (Figure 1B and Furlan et al., 2016). In the brain, a single band at around 83 kDa was also detected. The second antibody, C3868 (MC), showed reactivity at 83 kDa and at around 74 kDa in the brain, similarly to that found in the heart and in muscle homogenates. The MC antibody did not react with the 58-kDa isoform. Signal specificity was tested by omitting anti-Casq antibodies in a control immunoblot. As reported in Figure 1, (anti-rabbit) signals were absent.

A distinctive feature of Casq1 and Casq2 is the *N*-glycosylation consensus sequence (Asn-*X*-Ser/Thr) at the C terminus. An analysis of zebrafish Casq sequences by NetNGlyc^2^ and GlycoEP^3^ webservers identified a consensus sequence in position Asn336 (NVT) that is highly conserved in Casq2 among different species. In order to check the N-glycosylation state of native zebrafish Casqs, we performed digestion with N-glycosidase F on skeletal muscle and brain Casq2-enriched fractions. As shown in Figure 1C, after N-glycosidase F treatment, Casq2 apparent molecular weight was shifted (about 3 kDa) both in the skeletal muscle and in the brain, suggesting a native glycosylated form of Casq2 in both tissues, whereas Casq1a and Casq1b (detectable in muscle fractions) did not change mobility as they lack a specific consensus sequence. Taken together, mRNA analysis, immunological, and glycosylation data indicate that more than one Casq isoform is expressed in the brain.

### Identification of Casqs in Subcellular Fractions of Zebrafish Brain

**Figure 2:**
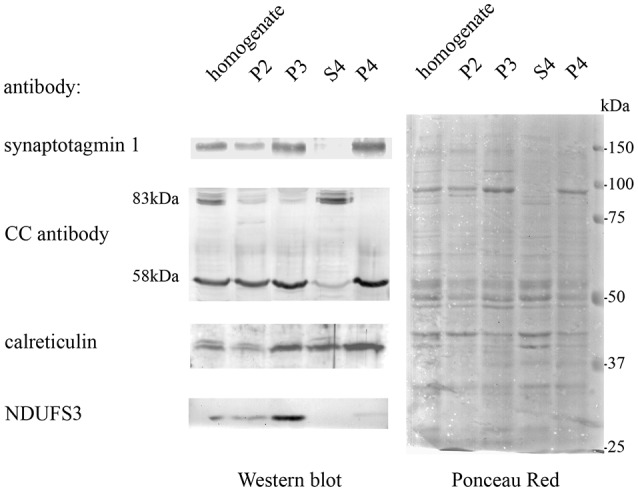
Adult brain subcellular fractionation: immunochemical profile. Immunoblot analysis of brain subcellular fractions. Equal protein amounts (40 μg) from each fraction were analyzed with CC antibodies to recognize Casqs and with antibodies specific for synaptotagmin1 (SYT1), calreticulin, NDUFS3 (as described in “Materials and Methods” section). Raw data derived from densitometric analysis are presented in Supplementary Table S1.

In order to obtain protein fractions enriched in Casq, differential centrifugation was performed on total brain homogenates derived from pooled adult zebrafish brains. Three membrane fractions (P1-2, P3, and P4—the latter being the lightest of the three) and a supernatant fraction S4 were obtained as described in “Materials and Methods” section. The different sub-fractions were characterized by specific immunological markers. As shown in Figure 2, synaptotagmin1 (SYT1), an abundant integral membrane protein of synaptic vesicles, was clearly identified in all membrane-containing fractions. Densitometric analysis (see Supplementary Table S1) showed an enrichment of the synaptotagmin signal in P2, P3, and P4 of 10.7, 17.6, and 26.1-fold, respectively, in comparison with S4, indicating that P4 was significantly enriched in membranes of synaptic origin. On the contrary, a mitochondrial marker protein associated to the inner mitochondrial membrane and matrix, NADH dehydrogenase [ubiquinone] iron-sulfur protein 3 (NDUFS3; Dieteren et al., 2008), was fully recovered in the P3 fraction, intermediate-speed pellet fraction, confirming the enrichment in mitochondria. Calreticulin was found in P4 pellet but was also abundant in the soluble fraction S4 according to previous reports (Holaska et al., 2001; Labriola et al., 2010). The distribution of Casq isoforms was not homogeneous among the fractions: the 58-kDa isoform, identified by CC antibody, was found in all membrane fractions and especially enriched (5.1-fold) in P4 in comparison to S4. On the contrary, the 83-kDa isoform was enriched in fraction S4 (6.9-fold) in comparison to P4. Finally, the 74-kDa isoform Casq1b, previously identified in the skeletal muscle, was not detectable in the brain fractions by immunoblotting. In conclusion, we obtained two fractions enriched in both the 58- and the 83-kDa proteins.

**Figure 3:**
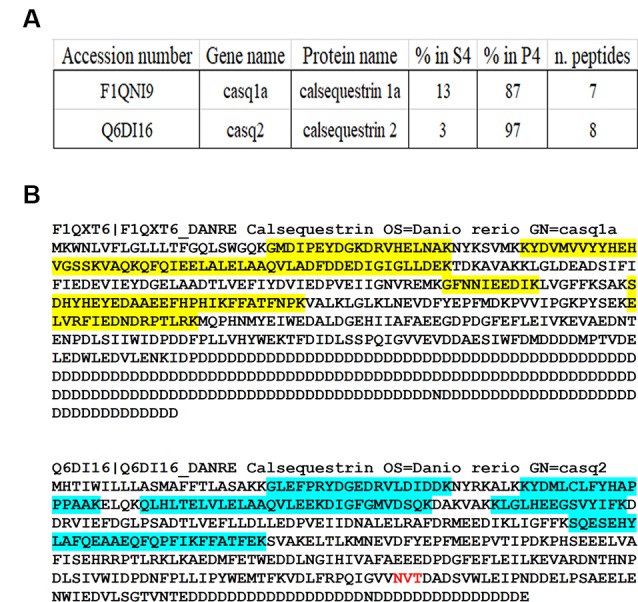
Identification of Casqs in adult brain fractions. **(A)** Relative abundance of the two detected isoforms. **(B)** Mapping of peptides identified by mass spectrometry on Casq sequences. In red is the consensus site for N-glycosylation.

Quantitative MS-based proteomics was applied to P4 and S4 fractions to confirm the identity of Casqs. Both isoforms were identified by several peptides spanning 9% of Casq1 and 22% of Casq2 (Figure 3B). In the P4 fraction, Casq2 was more abundant than Casq1a since the respective ranking by cumulative abundance was 1,074 for Casq2 and 1, 360 for Casq1a in 3,916 proteins (see Supplementary Table S2). Conversely, Casq1a was more abundant than Casq2 in S4, where the two isoforms ranked 1,910 and 2,141, respectively. No peptide from Casq1b was detected, either in S4 or in P4 fractions. Consistent with SDS-PAGE analysis, the MS results confirm the identification of Casq1a and Casq2 and suggest a different cellular compartmentation of the two isoforms.

### Casq mRNA Is Expressed in Multiple Brain Regions

**Figure 4:**
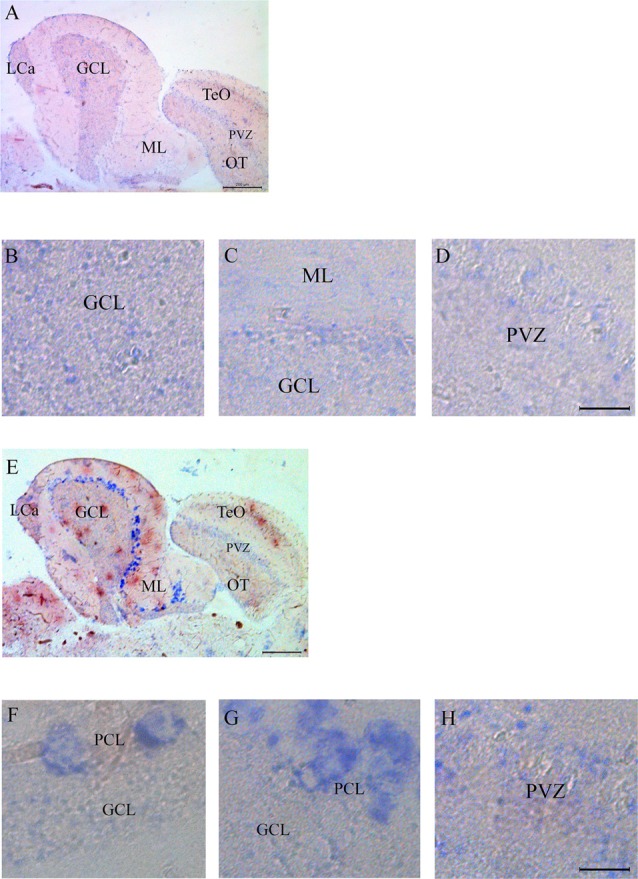
*In situ* analysis of *casq1a* [**(A–D)** and casq2 panels **(E–H)**] transcripts in sagittal brain sections (**A,E**, rostral to the right). Whole-brain sections and control sections are presented in Supplementary Figure S1. Anatomic terminology was inferred from Ullmann et al. (2010). CCe, corpus cerebelli; GCL, granule cell layer; Lca, caudal lobe of cerebellum; ML, molecular layer; PCL, Purkinje cell layer; PVZ, periventricular zone of optic tectum; TeO, optic tectum. **(A,E)** Bar: 200 μm. **(B–D,F–H)** Bar: 20 μm.

Expression of Casq RNA was studied by *in situ* hybridization. Isoform-specific riboprobes for *casq1* and *casq2* genes were used to examine whole sagittal sections, although focus was placed on the cerebellum and the optic tectum regions. In Supplementary Figure S1, sections of the whole brain processed with anti-sense RNA for *casq1* and *casq2* are shown paired with a control experiment performed without the riboprobes. Figure 4A shows the expression pattern of *casq1a* mRNA in the cerebellum and the optic tectum (TeO). Hybridization signals with casq1a riboprobe were found in the granule cell layer (GCL) of the cerebellum and the torus longitudinalis and in the stratum periventriculare (SPV) of the optic tectum. Higher magnification images of these regions are illustrated in Figures 4B–D that show a blue signal compatible with densely packed granular cells and absent in the control experiments (Supplementary Figures S1D–F). Figure 4E shows the expression pattern of *casq2* mRNA in a parasagittal section serial to that of Figure 4A. Casq2 mRNA was detected in the cerebellum, with a strong blue signal at the Purkinje cell layer and a weak signal in the optic tectum (SPV). Higher-magnification images show a strong signal at the level of the Purkinje cell bodies (panel F and G) and a weaker labeling in TeO SPV (panel H). In summary, these results indicate that the granule cells of the cerebellum and the optic tectum express Casq1, whereas the Purkinje cells of the cerebellum express Casq2.

### Differential Localization of Casqs in Zebrafish Cerebellum and Optic Tectum

**Figure 5:**
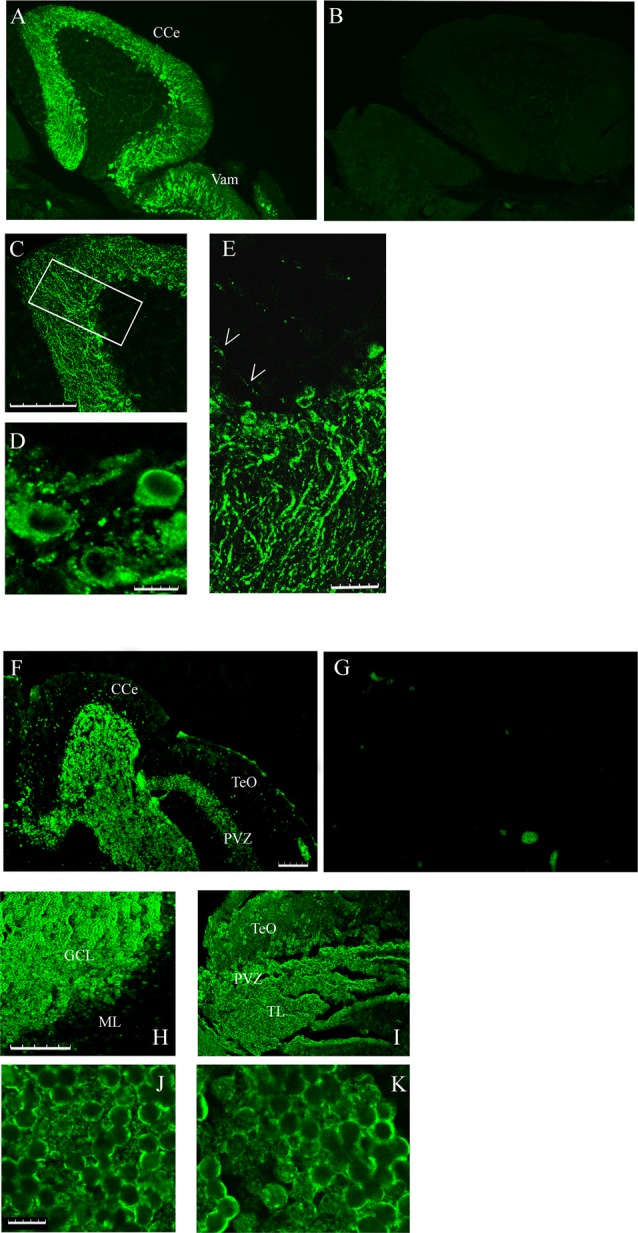
Immunofluorescence staining of sagittal brain sections area restricted to the cerebellum and the optic tectum (rostral to the right) decorated with CC **(A,C–E)** and MC **(F,H–K)** antibodies. **(B,G)** Images collected from control sections (see “Results” section) and acquired with the same conditions as in **(A)** and **(F)**, respectively. **(A,B,F,G)** Epi-fluorescence signals. **(C–E,H–K)** Images obtained by single optic section of confocal analysis. The box in **(C)** indicates the area shown at higher magnification in **(E)**. **(D)** PCL in a different region and focal plane. Arrows: axons of Purkinje cells. The abbreviations are the same as in Figure 4. **(A,C,F,H,I)** Bar: 100 μm. **(E)** Bar: 25 μm. **(D,J,K)** Bar: 7.5 μm.

**Figure 6:**
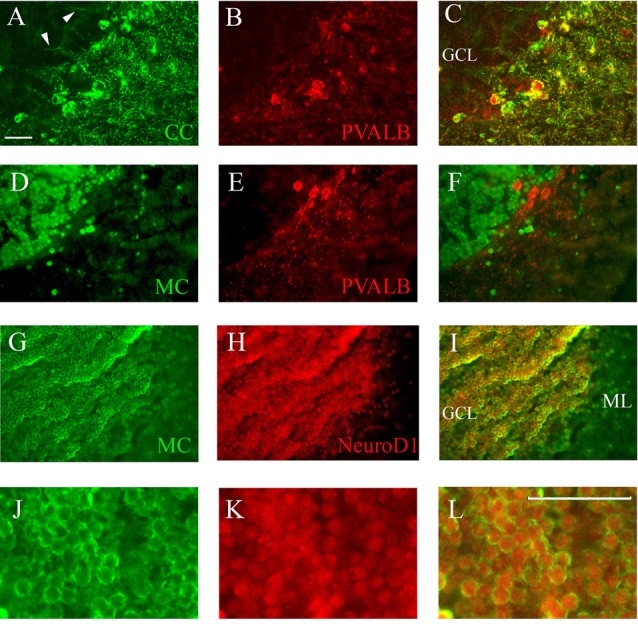
Co-staining of cerebellum with Casq and neuron-specific markers. **(A–C)** Epi-fluoresence signal obtained by double immunofluorescence with CC antibody (green) and anti-parvalbunin antibodies (red) in the cerebellum area. **(C)** A merge of the two images. Arrowheads indicate the axons of Purkinje cells. **(D–F)** Epi-fluoresence images obtained by double-staining with MC antibody (green) and anti-parvalbunin antibodies (red). **(D)** A merge of the two images. **(G–I)** Confocal images obtained by double-staining with MC antibody (green) and anti-NeuroD1 antibody (red). **(J–L)** Higher magnification of granule cells in the central area of the cerebellum double-labeled with MC (green) and anti-NeuroD1 antibody (red). Bar: 25 μm.

The different localization of *casq1* and *casq2* RNA in zebrafish brain suggested by *in situ* hybridization raises the intriguing possibility that Casq1a and Casq2 might be expressed in distinct cells. Cellular localization was investigated in parasagittal brain sections by immunofluorescence with MC and CC antibodies that recognize Casq1 and Casq2, respectively. With the CC antibody, a lively reaction was detectable in the cerebellum area (both corpus, CCe and valvula cerebelli; Val–Vam), being more intense at the level of the Purkinje cell layer and the molecular layer (ML; Figure 5A). Signal specificity was confirmed by processing a brain section with the same immunofluoresence protocol but excluding the CC antibody (Figure 5B). Confocal analysis (Figures 5C–E) of the fluorescence pattern showed, at higher magnification, drop-shaped large neurons located between the ML and the GCL, heavily stained in the cell bodies, except the nuclei, and organized in simple and in multiple layers according to the Purkinje cell distribution in the zebrafish cerebellum (Miyamura and Nakayasu, 2001). Dendrites extending into the ML of the cerebellum were also clearly stained with a punctuate pattern (E). In Purkinje cell bodies (D), a patchy reticulate pattern characteristic of Casq was clearly detectable. The granule cells were not stained (Supplementary Figure S2). Rare, thin, and dotted fluorescence, organized in a linear arrangement, was also detectable in the GCL (Figure 5E; see also Figure 6) in continuity with positive cell bodies. These linear structures are similar to the axons of chicken and mammal Purkinje cells (Villa et al., 1991; Sacchetto et al., 1995; Koulen et al., 2000). In cerebellum circuits of mammals and birds, the Purkinje cells send inhibitory projections (axons) to the deep cerebellar nuclei (DCN) whereas in zebrafish Purkinje the cell axons target eurydendroid cells, which are equivalent to mammalian DCN. The zebrafish Purkinje axons are shorter than the mammalian ones since the eurydendroid cells are big parvalbumin 7-negative neurons located in the granular layer in proximity to the Purkinje cells (Bae et al., 2009). A fluorescence pattern, similar to the CC pattern described above, was previously observed for ITPR1-positive Purkinje cells in zebrafish brain (Koulen et al., 2000), strongly suggesting that the CC-positive cells are Purkinje neurons. Additional parasagittal brain sections were processed by immunofluorescence with the MC antibody that recognizes exclusively Casq skeletal isoform in zebrafish muscle. The MC antibody (Figure 5F) strongly reacted at the cerebellum areas, mainly localized to the GCL and at the SPV of the optic tectum. The specificity of the signal was confirmed by processing a brain section with the same immunofluoresence protocol but excluding the MC antibody (Figure 5G). At higher magnification (Figures 5H,I), a reaction was clearly detectable in the peripheral area of granule cells. The confocal images of the cerebellum and the optic tectum SPV showed granule cells stained in the perinuclear area (Figures 5J,K). Some positive cells were also detected at the ML.

Identification of Casq-positive neurons was carried out by immunofluorescence in sagittal sections double-labeled with antibodies for cell-specific markers. Double immunofluorescence with specific parvalbumin (Pvalb) and MC antibodies (Figures 6A–C) showed that all neurons stained by CC were also Pvalb-positive, particularly at cell bodies and at proximal dendrites. Since Pvalb is a well-known marker for zebrafish and teleost cerebellum (Alonso et al., 1992; Takeuchi et al., 2015), these results show for the first time that the Purkinje cells of zebrafish cerebellum express Casq. Since in the western blot of brain homogenates the CC antibody identifies Casq2 (Figure 1B), the Purkinje cell isoform is a *bona fide* Casq2. The molecular layer area was further analyzed by confocal microscopy at higher magnification, confirming the differential localization of Casq1 and Casq2 proteins (see Supplementary Figures S3, S4). On the contrary, double immunofluorescence with MC and Pvalb antibodies showed an overall separation of the two signals (Figures 6D–F), indicating that the MC antibody decorated granule cells only. Using double immunofluoresence with specific antibodies for Casq1 (MC) and NeuroD1, a specific nuclear marker of granule cells in adult zebrafish (Takeuchi et al., 2015), Figures 6G–I shows that the NeuroD1-positive cells in the GCL are also clearly stained by the MC antibody in the perinuclear area where the ER is densely packed. A similar cellular co-localization of the immune signal was detectable at the granular cells of the optic tectum (PVZ) and the torus longitudinalis (not shown). Since the MC antibody does not recognize Casq2, our interpretation is that the protein identified in the granule cells is a *bona fide* Casq1.

### Identification of Casqs and Other Ca^2+^ Store Markers by Mass Spectrometry

Quantitative MS-based proteomics on subfractions P4 and S4 provided 24, 396 peptides corresponding to 3, 966 proteins. The corresponding MS data files were deposited in the ProteomeXchange Consortium *via* the PRIDE partner repository with the dataset identifier PXD015577. The list of proteins identified by more than one unique peptide (2, 886 proteins) is provided in Supplementary Table S2. General neuronal markers such as NSE (eno2) expressed in mature neurons and glia cells (Bai et al., 2007), MAP2 microtubule-associated protein, calbindin 2, and neurofilament light polypeptide b (Neflb) were identified. Moreover, neuronal type-specific markers such as Grid2 and Ca8 (carbonic anhydrase 8), proteins known to be selectively expressed in the Purkinje cells of the cerebellum (Huang et al., 2014), calbindin 2, marker of dendrites from Purkinje and granule cells, slc17a6a and slc17a6b (vesicular glutamate transporter 2.1 and 2.2) and slc17a7a (Vglut1) markers of granular layer, Lca (locus caudalis cerebelli), and torus longitudinalis glutamatergic neurons. Finally, a marker for Bergmann glia, Slc1a3b (Bae et al., 2009), was also identified. Trans-golgi network proteins Lman1 (ERGIC53) and EMC3 were present, whereas abundant proteins from mitochondrial membranes, such as cytochrome c oxidase I and II, were not detected in P4 and S4, suggesting a negligible contamination of the mitochondria.

### Overall Protein Distribution in P4 Membrane Pellet vs. S4 Supernatant

A manually curated list of informative proteins identified in P4, S4, or both is presented in Supplementary Table S3. Three protein groups display a different partition between P4 and S4: the first group is comprised of soluble cytoplasmic proteins 95–100% enriched in S4 (for example, enolases), the second group consists mostly of trans-membrane proteins (belonging to pre- and post-synaptic membranes, ER or Golgi membranes, and plasma membrane) 95–100% enriched in P4, and the third group of proteins is more homogeneously distributed between P4 and S4, such as ER/Golgi resident luminal proteins Grp94, PDI, and calreticulin, which was found both in P4 (53%) and in S4 (47%) according to western blot analysis (Figure 2). Mass spectrometry identified another ER resident protein calnexin (91% in P4). Calnexin and calreticulin are two ER chaperon proteins that share a common sequence and structure, but calnexin is membrane-bound and calreticulin is soluble according to their differential compartmentation between P4 and S4. Proteins belonging to various vesicles were also identified; in particular, clathrin-coated vesicle components were restricted to P4 fraction and COPI vesicle components were restricted to S4. Several synaptic proteins were found exclusively in the P4 fraction, among them, peptides belonging to vesicular glutamate trasporter 1, synaptotagmin Vb, VAMP2, and ionotropic glutamate receptor. In addition, MAP2, microtubule-associated protein 2 (E7FBI2), described as a component of dendritic spines (Izant and McIntosh, 1980), was identified in the P4 fraction by 16 peptides. Two important calcium store markers, the IP3-sensitive Ca^2+^ channel type 1 (ITPR1) and an isoform of sarco-endoplasmic reticulum Ca^2+^ pump (ATP2a2a), were identified in fraction P4. No mitochondria membrane protein was detected both in P4 and in S4, further suggesting the lack of substantial contamination by mitochondria. Global Gene Ontology enrichment analysis (see Supplementary Figure S6) confirmed that the majority of either membrane or membrane-associated proteins were enriched in the P4 fraction.

## Discussion

In this article, we studied Casq expression and distribution in the adult brain of zebrafish with particular focus on the cerebellum and the optic tectum. Our aim was to determine how many Casq isoforms are expressed and to study their cellular distribution and subcellular localization. Due to the scarcity (in comparison with mammals) of immunomarkers for adult zebrafish tissues, we used multiple approaches to integrate a different set of data. Thus, we *in situ* hybridization with Casq-specific designed primers, single or double immunofluorescence experiments, with commercially available antibodies which recognize zebrafish skeletal muscle Casq1 and Casq2, and mass spectrometry.

The major findings are as follows: (1) Casq1a and Casq2 are expressed in zebrafish brain especially in the cerebellum and the optic tectum; (2) Casq1b is not detectable; (3) Casq1a is expressed in granule cells of the cerebellum and the optic tectum; and (4) Casq2 is concentrated in Purkinje cells at cell bodies, axons, proximal and distal dendritic shafts.

### Identification and Localization of Casqs

#### Comparison With Other Teleostei and Mammals

Among teleostei, in *Solea senegalensis*, four mRNAs with different organ specificity were identified: *casq1a* and *casq1b* mainly expressed in the skeletal muscle, *casq2a* in the heart, and *casq2b* in the brain of juvenile fishes (Infante et al., 2011). Solea Casq2b presents 69.9% identity with zebrafish Casq2 (protein ID: Q6DI16). In mammalian brain (mouse), *Casq2* RNA is expressed in the Purkinje cells of the cerebellum (Pavlidis and Noble, 2001; Rong et al., 2004). In this article, we show for the first time that Casq1 mRNA and protein are expressed in the granular cells of the cerebellum and the optic tectum. *In situ* hybridization showed an isoform-specific pattern of Casq1a mRNA expression in the cerebellum, in the GCL of corpus and valvula cerebelli, and, interestingly, in the optic tectum–torus longitudinalis granular cells which belong to specific neuronal circuits defined as cerebellum-like structures. Optic tectum and torus longitudinalis are cerebellum-like structures consisting of a molecular layer (OTML), a principal cell layer (type I neurons), and a granular structure (TL) composed of densely packed glutamatergic neuronal cell bodies. The TL cells were positive with MC antibody as PVZ, while the type I neurons of the SFGS stratum were negative. Another cerebellum-like structure, medial octavolateral nucleus, crossed by projections of eminentia granularis cells which project their dendrites in the molecular layer of crista cerebellaris (Bell et al., 2008; Robra and Thirumalai, 2016), has not been analyzed. The localization of Casq1 protein is in agreement with *in situ* mRNA localization. The MC antibody is the commercially available polyclonal antibody that detects Casq1 in zebrafish skeletal muscle by both western blot and immunofluorescence (see Furlan et al., 2016). Validation of the antibody was performed previously in the skeletal muscle, where Casq1 is extremely abundant, by correlating the characteristic chemical properties of Casq1 (Stains’s all staining and Ca^2+^-induced shift in SDS-PAGE) with immunodetection. The reactivity of the antibody was weak in western blot but reliable and specific for the Casq1 isoform compared with Casq2 in zebrafish muscle. For these reasons, we found a low intensity of the western blot signal in the brain by MC antibody but with good isoform specificity and high signal-to-noise ratio, as shown in Figure 1. The possibility that the MC antibody cross-reacted with a brain-specific protein similar to Casq1 but different from it cannot be excluded, but it is unlikely since Casq1a was unequivocally identified in brain homogenates by mass spectrometry.

By *in situ* hybridization and immunofluorescence, we show for the first time that Casq2 mRNA and protein are expressed in zebrafish cerebellum Purkinje cells of both corpus and valvula cerebelli. In particular, CC antibody reactivity implies that the expression of Casq2 extends from the cell body to the dendrites and the axons. It is ruled out that the signal refers to cross-reactivity with Casq1 because it is not detectable in Purkinje cell dendrites by Western blot and immunofluorescence. The strong *in situ* hybridization and immunofluorescence signals in Purkinje cells indicate a high concentration of the protein. A similar concentration in Purkinje cells has been observed in chicken (Villa et al., 1991; Takei et al., 1992), where Casq is a component of specialized ER sub-domains distributed along all Purkinje regions, except the majority of the dendritic spines.

### Differential Cellular Localization of Casqs: Physiological Implications

*In situ* hybridization and immunofluorescence experiments show that Casq1 and Casq2 are differentially localized in zebrafish brain with virtually no overlap. We found two main differences: (a) the high concentration of both Casq2 protein and mRNA in Purkinje cells as compared to other neurons; and (b) the significant expression of Casq1 in the granular cell layer of the cerebellum and the optic tectum.

Here, we show for the first time the identification and the localization of the skeletal Casq1 isoform (both RNA and protein) in the granular cells of a vertebrate cerebellum. A characteristic of this species is the continuous renewal of some neurons such as granule cells, Bergmann glia, and inhibitory interneurons (Grandel et al., 2006; Kani et al., 2010; Jászai et al., 2013; Kaslin et al., 2013). These cells regenerate in specific areas and, during cell differentiation, migrate to the final functional region (Zupanc et al., 2005). We found the highly positive Casq1 cells to be sparse in the molecular layer of adult cerebellum where migrating granules transit (see also Supplementary Figure S5E). It appears that the neuronal migration and the proliferation of granule cells is regulated by Ca^2+^ release from ER stores *via* IP3- and/or ryanodine-sensitive channels (Kumada and Komuro, 2004; Komuro et al., 2015; Horigane et al., 2019). It is plausible that Casq1 plays a role in such a mechanism in fish, especially in shaping either Ca^2+^ transients or spikes, as occurs in the skeletal muscle (Tomasi et al., 2012). Preliminary evidence of Ryr1 positive cells in the molecular layer are presented in Supplementary Figure S5F. In this perspective, Casq1 could be a functional marker of regenerating and/or migrating neuronal progenitors in adult and possibly developing zebrafish.

Neuronal plasticity mechanisms, such as LTD, have not been described in zebrafish. Little is known on the molecular composition of Ca^2+^ stores in the Purkinje cell of zebrafish cerebellum. As for ER Ca^2+^ channels, the RNA of five ryanodine receptor genes is expressed in zebrafish brain (Darbandi and Franck, 2009, and Supplementary Figure S5F); in Purkinje cells, the ITPR1 protein has been identified (Koulen et al., 2000). We found that Casq2 is concentrated in all cellular compartments (axon, cell body, and dendrites) of Purkinje cells (as previously shown for chicken cerebellum; Volpe et al., 1991) similarly to what happens for ITPR1 (Koulen et al., 2000), differently to cell homogeneous distribution of Calreticulin and SERCA (Supplementary Figures S5A,B,G,H). In zebrafish, like in mammals, a high concentration of cytoplasmic Ca^2+^-binding proteins in Purkinje cells implicate a high buffering power in the cytosol (Supplementary Figure S5D): zebrafish Casq2 could be essential in maintaining the high-capacity and strictly localized Ca^2+^ stores in the ER despite cytoplasmic buffering. Store operating calcium entry (SOCE) is the Ca^2+^ refilling mechanism of ER in the granular and the Purkinje cells of mammals (Hartmann et al., 2014; Ryu et al., 2017; Wegierski and Kuznicki, 2018). The acidic C-terminal of mammal Casq1 is involved in the mechanism of SOCE (Wang et al., 2015; Zhang et al., 2016). In mammalian brain, stromal interaction molecule 1 (STIM1) has been shown to link mGluRs and IPTR1 signals and to play a critical role in cerebellar Purkinje cells (Hartmann and Konnerth, 2008); moreover, STIM and Orai have been identified in several neuronal compartments (Segal and Korkotian, 2014). Nothing is known about SOCE in zebrafish brain except for the positive expression of STIM1 in the neuronal progenitor cells (Tse et al., 2018). We obtained preliminary evidence of STIM1 expression in Purkinje cells and in granule cells, although at lower levels (see Supplementary Figure S5C), suggesting that SOCE could occur and that both Casq1 and Casq2, having a highly acidic C-terminal tail, could be involved in its regulation.

Given that the different Casq isoforms are preferentially expressed in distinct cell types, our results suggest that they might have very different functions in the zebrafish brain. This might, at least in part, be referable to the long acidic tail at the C terminus of zebrafish Casq1a. Structural studies on mammal Casq show that the C-terminal tail in domain III is an intrinsically disordered region involved in the polymerization and the binding of cations and transition metals (Bal et al., 2011). It is plausible to speculate that these protein domains could bind neurotossic transition metals in particular environment conditions. Thus, in aquatic organisms, the maintenance of a Casq isoform with a very long acidic tail could have conferred an evolutive advantage.

## Data Availability Statement

The datasets generated for this study have been deposited to the ProteomeXchange Consortium *via* the PRIDE partner repository with the dataset identifier PXD015577.

## Ethics Statement

The animal study was reviewed and approved by the University of Padova Ethical Committee on Animal Experimentation and Ministero della salute (Project Number D2784.N.BGL).

## Author Contributions

AN and SF contributed to the conception, the design of the study and drafted and wrote the manuscript. MC, MM, SF, and SM performed the experiments and analyzed the data. FA and PV critically revised the manuscript. All the authors contributed to manuscript revision and read and approved the submitted version.

## Conflict of Interest

The authors declare that the research was conducted in the absence of any commercial or financial relationships that could be construed as a potential conflict of interest.

## Footnotes

^1^http://www.perseus-framework.org

^2^http://www.cbs.dtu.dk/services/NetNGlyc

^3^https://webs.iiitd.edu.in/raghava/glycoep/submit.html

## Abbreviations

Casq: calsequestrin protein
*casq*: calsequestrin gene and RNA
CC antibody: antibody to canine cardiac Casq Pa1–913
CCe: corpus cerebelli
COPI: coatomer protein complex
DCN: deep cerebella nuclei
ER: endoplasmic reticulum
GCL: granule cell layer
Kd: dissociation constant
IP3: inositol triphosphate
ITPR1: inositol triphosphate-sensitive Ca^2+^ channel type 1
LCa: lobus caudalis cerebelli
LTD: long-term depression
MC antibody: antibody to mouse cardiac Casq C3868
ML: molecular layer
MON: medial octavolateralis nucleus
NeuroD1: neurogenic differentiation 1
OTML: optic tectum marginal layer
PCL: Purkinje cell layer
PDI: protein disulfide isomerase
SPV: stratum periventriculare
Pvalb: parvalbumin
*Ryr*: ryanodine-sensitive Ca^2+^ channels gene
S1, S2, S3, S4: surnatant fractions 1–4
P2, P3, P4: pellet fractions 2–4
SERCA: sarco-endoplasmic reticulum calcium pump
SOCE: store operating calcium entry
STIM1: stromal interaction molecule 1
TeO: optic tectum
TL: torus longitudinalis
Val-Vam: valvula cerebelli.
