# Supplementary material for: Calsequestrins New Calcium Store Markers of Adult Zebrafish Cerebellum and Optic Tectum
Source: Front Neuroanat. 2020 Apr 21;14:15. doi: 10.3389/fnana.2020.00015 (PMC7188384; doi:10.3389/fnana.2020.00015)
Supplement: Supplementary file 2 [file Data_Sheet_1.pdf]

Supplementary Information for “Calsequestrins new Calcium store markers of adult zebrafish cerebellum and optic tectum”

Supplementary Figure S1.

In situ hybridization. Whole brain sections were incubated with antisense probe for Casq1 (a), Casq2 (b) and without probe (c). Analysis reported in the paper (Figure 4) was focused on the boxed areas in a and b. Panels a, b, c Bar 500µm; panels d, e, f Bar 20 µm.

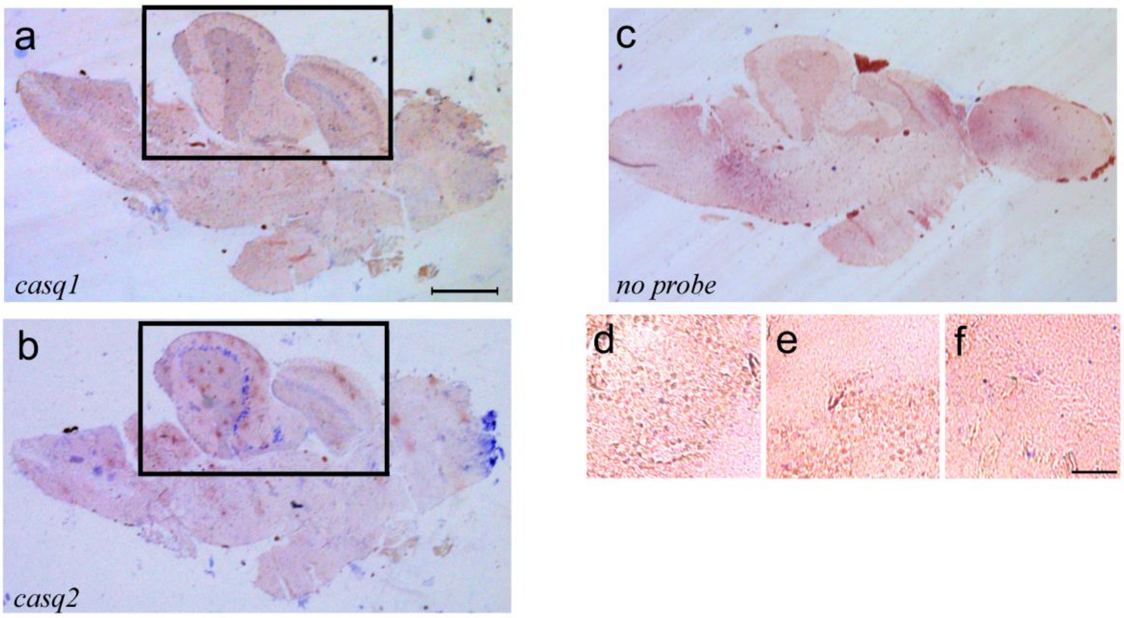

Supplementary Figure S2.

Confocal analysis of granule cells of cerebellum. Granule cells evidenced by DAPI (blue) (in panel b) are negative with anti Casq2 (CC antibody, green, panel a). a Purkinje cell (asterisk) with saturated signal is visible Bar 7,5 µm

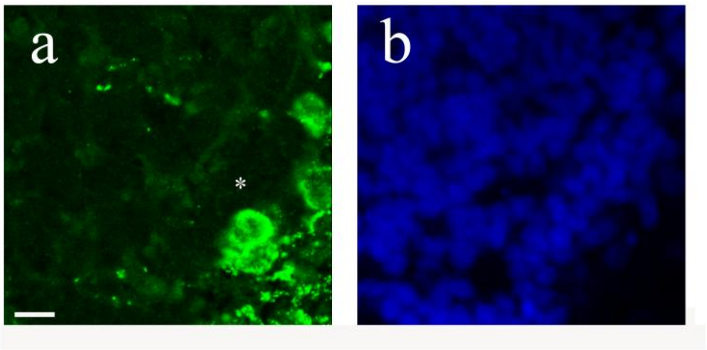

Supplementary Figure S3.

Confocal analysis of dendritic shafts double labeled for calsequestrin and parvalbumin.

Representative images of cerebellum molecular layer stained by MC (panel A) and anti Parvalbumin (panel B) antibodies, show positive granular cells (panel A asterisks) and a negative Purkinje cell (PC). In the merge image (panel C) MC antibody revealed a fine punctuate pattern (green) randomly distributed within or outside the dendritic shaft (red), probably representing parallel fibres (granule cell axons) transversally sectioned negative for Pvalb. A Purkinje cell body (PC) is positive with anti Pvalb (red) and negative with MC (green).

With CC antibody (panel D) the fluorescence pattern shows a spotty appearance and is restricted to the dendritic shafts similarly to anti parvalbumin (panel E) pattern. CC positive spots were rounded or elongated structures distributed along the main dendritic axis. Some dendrites indicated by lower case letters in panels C and F were further analyzed for co-localization (see Supplementary Figure S4). Analysis was carried out in a Leica SP5 microscope. Scale bar: 15 μm

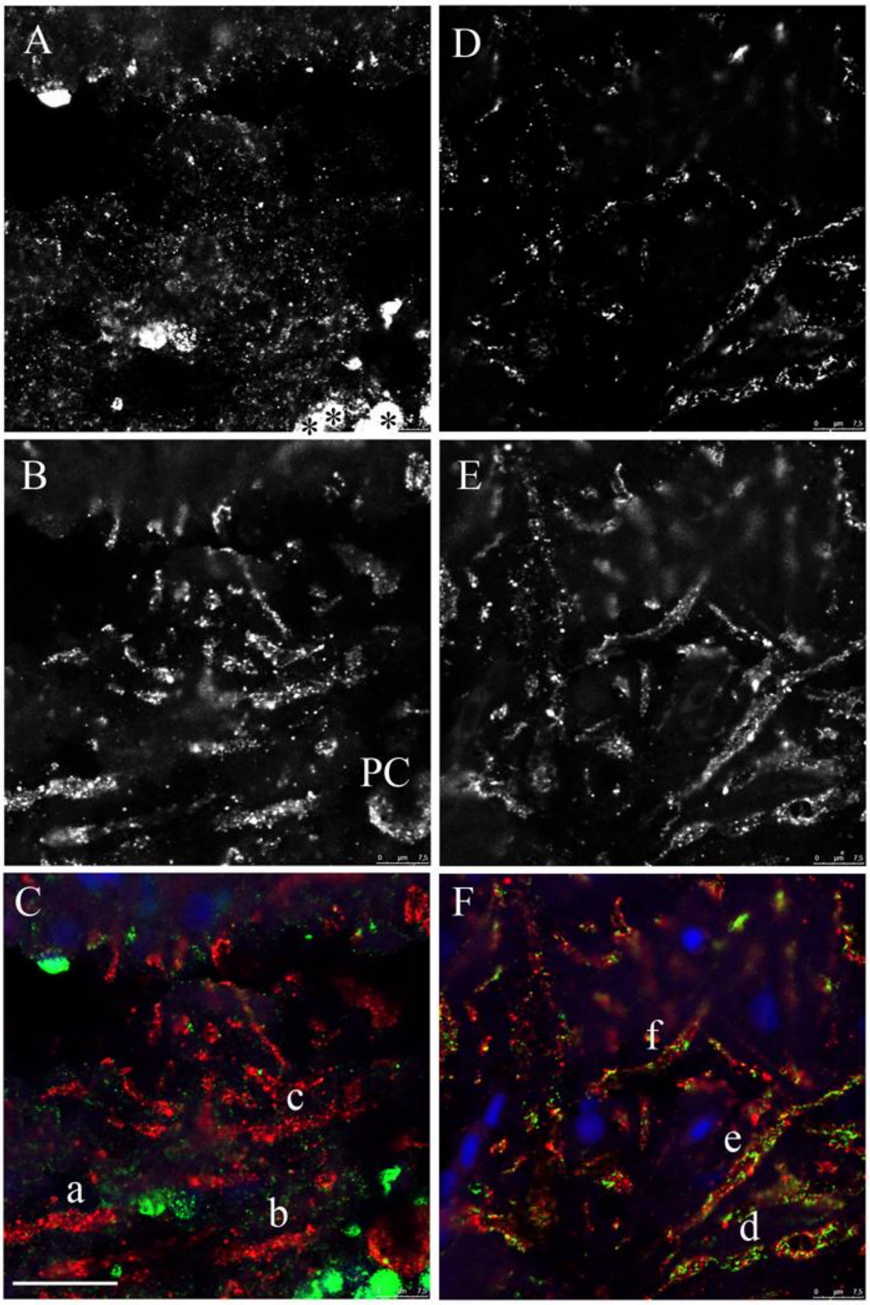

Supplementary Figure S4.

Co-localization analysis of dendritic shafts double labeled for calsequestrin and parvalbumin.

Dendrites of Purkinje cells decorated with CC antibody (green panels d, e, f), clearly show discrete puncta and reticular structures in proximity of the Pvalb signal (red) but the signal did not fully overlap. On the contrary MC fluorescence puncta (green, panels a,b,c) are located outside the Pvalb positive dendrites. Images were minimally processed using Photoshop (Adobe, Creative Suite 6) to adjust levels, contrast and brightness. Co-localization analysis was performed using Volocity 6.0 software (Perkin Elmer) and quantification was obtained on the different channels of the merged images and normalized against background. Global Pearson correlation coefficient was 0,039 in supplemental Figure S3 panel C and 0,412 in supplemental Figure S3 panel F. The mean Pearson correlation coefficient for the regions of interest ROIs shown in Supplementary Figure S4 was 0,185±0,037 for a, b, c and 0,168±0,038. for dendrites d, e, f.

In conclusion co-localization with Pvalb was not significant for both CC and MC antibodies as expected for proteins localized in different intracellular compartments and/or different cells.

Bar: 3,75 μm

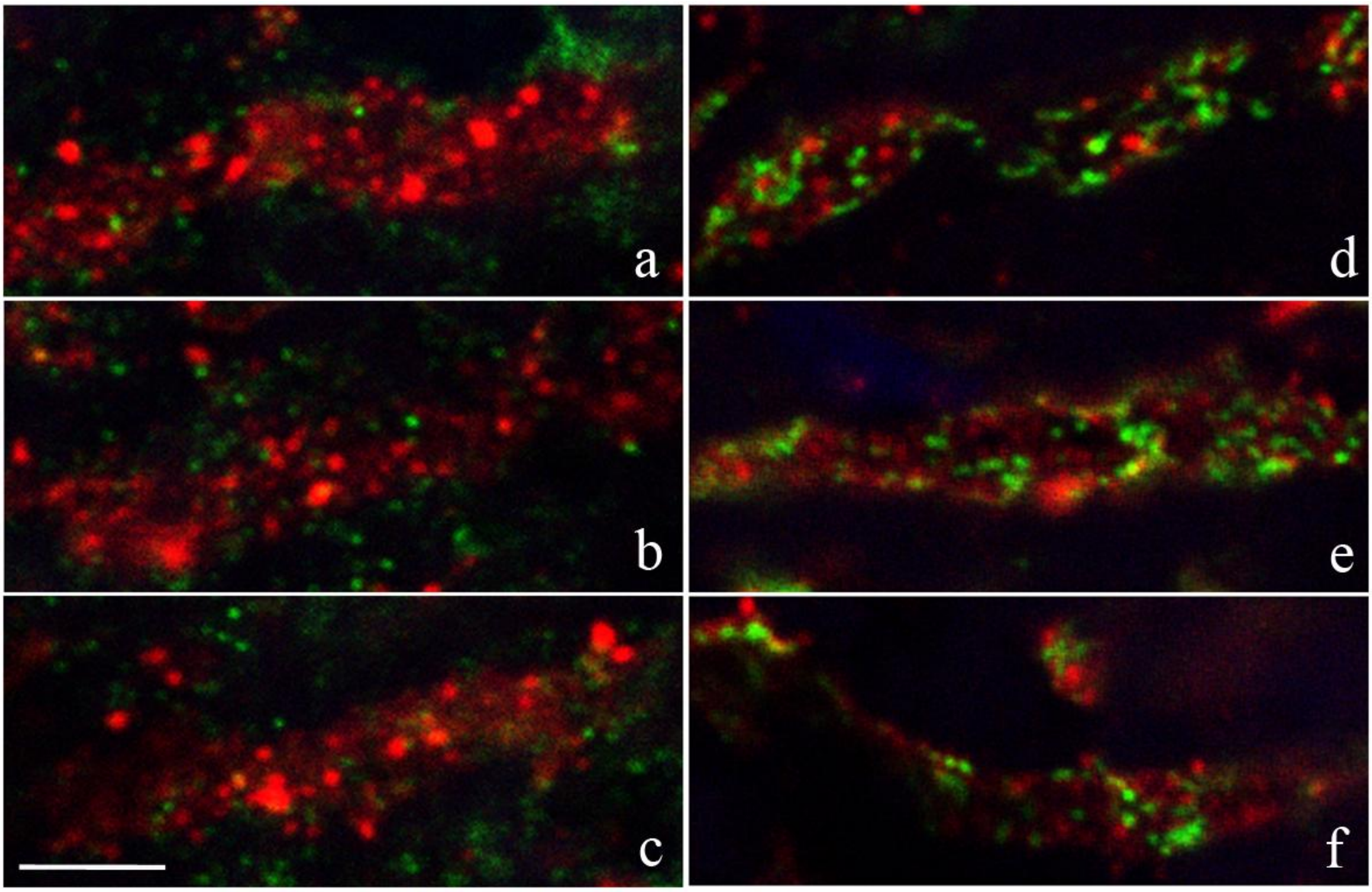

Supplementary Figure S5. Heterogeneous localization of Ca^{2+} store markers in cerebellum.

Immunofluorescence staining of parasagittal sections of corpus cerebelli area with anti Casq (panels A and E) and other Ca^{2+} store markers antibodies, as reported. All panels are oriented with GCL (granule cell layer) on the right, and ML (molecular layer) on the left. PCL: Purkinje cell layer. Antibodies to calreticulin (CALR), a well-known and widely expressed intra ER Ca^{2+} binding protein, display an homogeneous distribution between Purkinje and granule cells (Panel H) in comparison with Casq1, Casq2, and parvalbumin (panels E, A, D respectively). Two Ca^{2+} store markers ITPR1, and SERCA (sarco/endoplasmic reticulum ATPase atp2a1, atp2a2, atp2a3) were identified in this study by mass spectrometry enriched in P4 fraction.. As shown in panel G an anti SERCA 2 antibody shows a signal that is more intense at GCL, PCL and less intense at ML, while anti ITPR (panel B) heavily stains Purkinje cell bodies and ML with a reticulate pattern. In addition an anti Ryr1 antibody shows moderate reaction at PCL and ML with a cloudy pattern, instead of a reticulate one (Compare panel F with panels A-D). The different immunofluoresence patterns indicate heterogeneous expression of Ca^{2+} store markers between Purkinje and granular cells in contrast with a general ER marker such as calreticulin.Bar 25 μm.

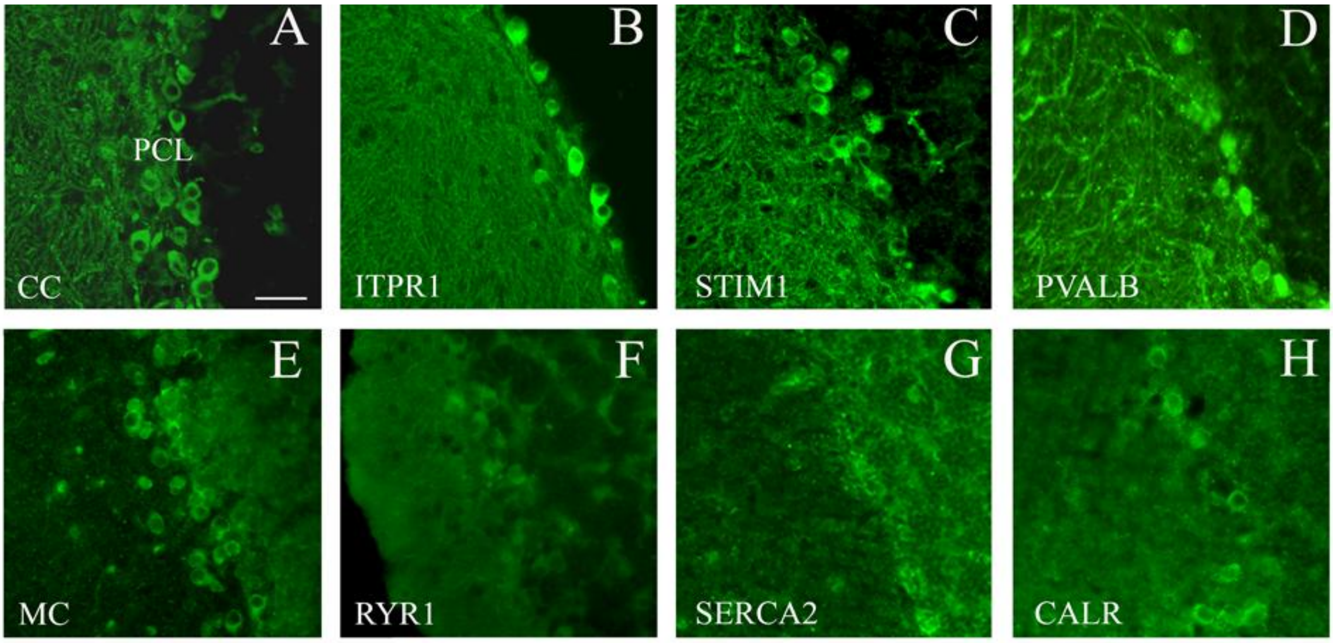

Supplementary Figure S6

Gene Ontology (GO) enrichment analysis of category counting based on Perseus Tool.

The Gene Ontology category “Cellular Component” (GOCC) was selected for this analysis because refers not to processes but rather to cellular anatomy. Panel A shows the number of proteins identified exclusively in P4 or in S4 belonging to the indicated classes. Classes that hit less than 1% of total identified proteins were not considered. Panel B shows the number of proteins belonging to the indicated classes with an intensity ratio (S4/P4) less than 0.5 (proteins enriched in P4) and intensity ratio (S4/P4) more than 2.0 (proteins enriched in S4). Classes that hit less than 1% of total identified proteins were not considered. These data show that the majority of proteins annotated in the specified classes is enriched in P4

A

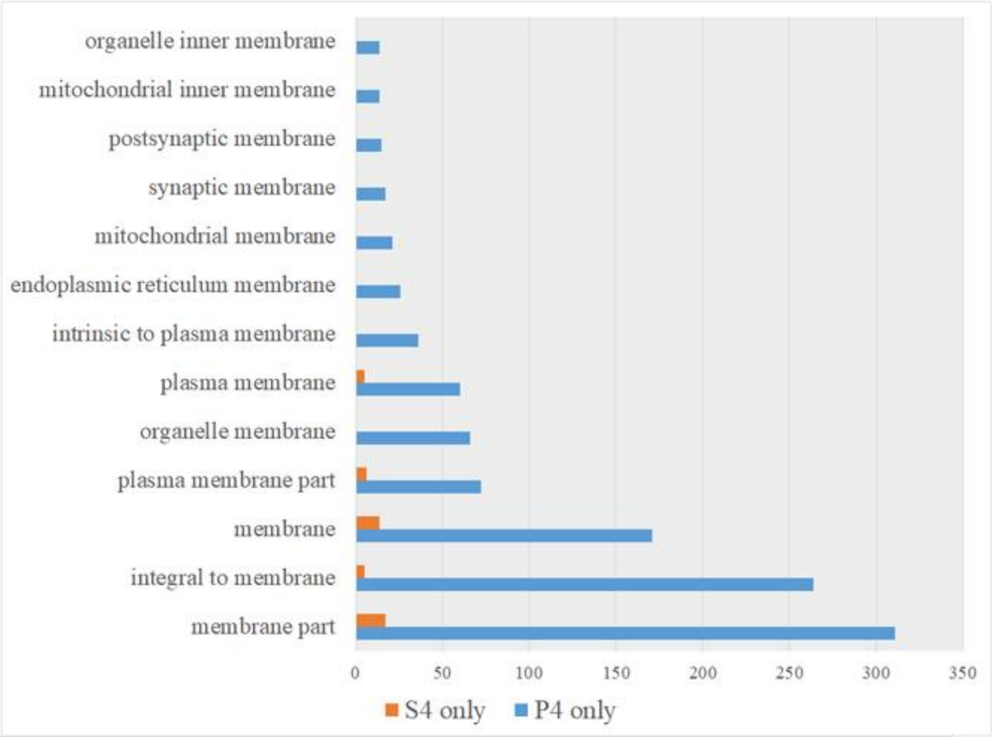

B

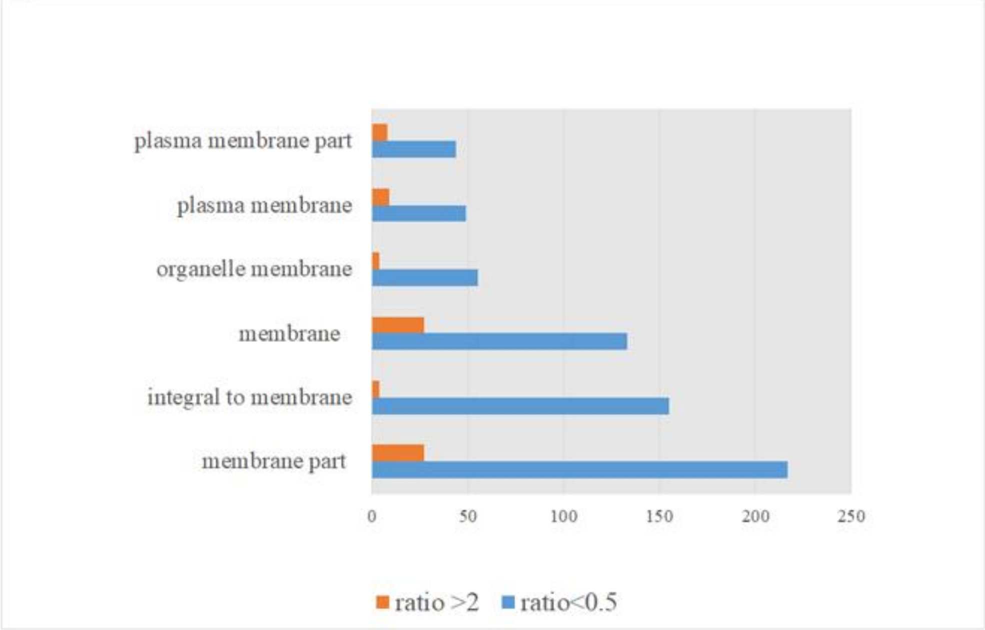

Supplementary Table S1. Densitometric analysis of Figure 2A

	P2 (O.D)	P3 (O.D)	S4 (O.D)	P4 (O.D)
synaptotagmin	541	1800	102	2662
calreticulin	2195	7622	6610	13949
calsequestrin 58kDa	953	943	382	1959
calsequestrin 83kDa	262	163	1488	214

Supplementary Table S2 on separate file Supplementary Table S2.xls

Supplementary Table S3. Partial, manually curated, list of proteins identified in S4 and P4 fractions by Mass spectrometry.

Proteins were grouped based on known subcellular localization. TM=transmembrane protein; MA= membrane-associated protein; S= soluble protein.

Accession number	Gene name	Protein name	Compartment	Protein type	% in S4	% in P4	n. peptides
A3QK31	slc17a7a (vGluT1)	Vesicular glutamate transporter 1	PRESYN	TM	2	98	4
E7FAK4	slc17a7b	Solute carrier family 17 member 7b	PRESYN	TM	0	100	3
F1QPP6	slc17a6a (vGluT2)	Vesicular glutamate transporter 2.2	PRESYN	TM	3	97	7
Q5W8I8	slc17a6b	Vesicular glutamate transporter 2.1	PRESYN	TM	2	98	9
Q504A0	syt5b	Synaptotagmin Vb	PRESYN	TM	0	100	13
B3DGS8	gria1a	Glutamate receptor, ionotropic, AMPA 1a	POSTSYN	TM	0	100	5
B0V2X4	gria2a (GluR2)	Glutamate receptor, ionotropic, AMPA 2a	POSTSYN	TM	0	100	4
F1QQC1	gria2b	Glutamate receptor, ionotropic, AMPA 2b	POSTSYN	TM	0	100	2
F1QA08, F1QBT6	gria4a;gria4b	Glutamate receptor, ionotropic, AMPA 4a/b	POSTSYN	TM	0	100	3
F1R366	grin1a	Glutamate receptor, ionotropic, N-methyl D-aspartate 1a	POSTSYN	TM	0	100	3
Q6ZM67	grin1b	Glutamate receptor, ionotropic, N-methyl D-aspartate 1b	POSTSYN	TM	0	100	2
Q6R005	dlg4 (PSD95)	Disks large homolog 4	POSTSYN	MA	73	27	3
F8W4B1	itpr1a	Inositol 1,4,5-trisphosphate receptor, type 1b	POSTSYN/SPINES	TM	0	100	8
B8JIC5	homer1b	homer scaffolding protein 1b	POSTSYN/SPINES	MA	76	24	10
F1R1T3	cacna1c	calcium channel, voltage-dependent, L type, alpha 1C subunit	SYNAPTIC	TM	0	100	3
A0A2R8QKE7	nrxn1a	Neurexin-1a	SYNAPTIC	TM	0	100	5
A0A2R8RZF2	cdh2	Cadherin-2	SYNAPTIC	TM	0	100	4
Q9DES8	glrba;glrbb	Glycine receptor betaZ subunit	SYNAPTIC	TM	0	100	2
E9QC31	glra1	Glycine receptor subunit alphaZ1	SYNAPTIC	TM	0	100	2
Q7ZV18	stx4	syntaxin 4A	SYNAPTIC	TM	0	100	4
E7F3U8	chrm4a	Muscarinic acetylcholine receptor	SYNAPTIC	TM	0	100	2
F1Q7F9	sypl2a	Synaptophysin-like 2a	SYNAPTIC	TM	0	100	2
Q503N6	syngr3a	Synaptogyrin	SYNAPTIC	TM	0	100	3
Q6PC44	calb2a	Calbindin 2a	SYNAPTIC	S	1	99	7
F1QU50	calb2b	Calbindin 2b	SYNAPTIC	S	0	100	5
A9C3Q5	atp2a2a	Calcium-transporting ATPase	ER	TM	0	100	3
Q6ZM60	atp2a2b	Calcium-transporting ATPase	ER	TM	66	34	17
Q1LY88	stim1a	Stromal interaction molecule 1a	ER	TM	0	100	9
F1QK54	canx	Calnexin	ER	TM	9	91	19
A5PMG4	ahcyl1 (IRBIT)	Adenosylhomocysteinase	ER	S/MA	71	29	6
Q58EG2	erlin1	Erlin-1	ER	TM	0	100	3
A3QK16	erlin2	Erlin-2	ER	TM	0	100	5
Q1ECX9	Pdia4	Protein disulfide-isomerase A4	ER lumen	S	1	99	20
B0S5S6	pdia5	Protein disulfide-isomerase A5	ER lumen	S	2	98	7
Q90WA5	pdia6	Protein disulfide-isomerase A6	ER lumen	S	99	1	13
Q6P3G9	erp44	Endoplasmic reticulum protein 44	ER lumen	S/MA	0	100	6
F1Q8W8	calr	Calreticulin	ER lumen	S/MA	47	53	12
Q6P3L3	hspa5 (GRP78)	Heat shock protein 5	ER lumen	S/MA	45	55	19
Q7T3L3	Hsp90b1 (GRP94)	Chaperone protein GP96	ER lumen	S/MA	56	44	32
F1R429	Lman1 (ERGIC53)	Lectin, mannose-binding, 1	ER/GOLGI	TM	0	100	4
Q7SXW4	emc3	ER membrane protein complex subunit 3	ER/GOLGI	TM	0	100	2
F1QDQ1	copa	Coatomer subunit alpha	vescicle	S/MA	2	98	6
Q66HV4	copb1	Coatomer subunit beta	vescicle	S/MA	100	0	12
B0R171	copb2	Coatomer subunit beta'	vescicle	S/MA	81	19	21
Q7SZE5;F1R7W8	sec23a;sec23b	Protein transport protein Sec23A	vescicle	S/MA	6	94	4
Q9PUE4	copg2	Coatomer subunit gamma-2	vescicle	S/MA	87	13	11
Q7ZW27	arcn1a	Coatomer subunit delta	vescicle	S/MA	100	0	6
Q7ZU89	arcn1b	Coatomer subunit delta	vescicle	S/MA	6	94	7
A8WFR0	lamp5	Lysosome-associated membrane glycoprotein 5	clathrin-coated vesicle	TM	0	100	2
Q7T2C6	rab7a	RAB7, member RAS oncogene family	cytoplasmic vesicle membrane	TM	0	100	2
Q7T070	rhbg	Ammonium transporter Rh type B	clathrin-coated vesicle	TM	0	100	2
A5PMM5	snx18a	Sorting nexin	clathrin-coated vesicle	TM	11	89	3
Q5XJP3	syngr1a	Synaptogyrin	clathrin-coated vesicle	TM	0	100	3
Q6IQK3, Q7SZZ5	vamp3;vamp2	Vesicle-associated membrane protein 2/3	clathrin-coated vesicle	TM	2	98	1
F1Q932	cttn	Cortactin	Cytosol	S	99	1	13
Q6PC12	eno1a	Enolase 1, (Alpha)	Cytosol	S	86	14	17
Q6PC89	eno1b	Enolase 1b, (alpha)	Cytosol	S	100	0	9
Q6GQM9	eno2	Enolase 2	Cytosol	S	92	8	14
Q6TH14	eno3	Enolase 3	Cytosol	S	98	2	14
Q5XJ10	gapdh	Glyceraldehyde-3-phosphate dehydrogenase	Cytosol	S	94	6	10
F1QNI9	casq1a	calsequestrin 1a			10	90	5
Q6DI16	casq2	calsequestrin 2			3	97	6
